# Nurr1 Is Not an Essential Regulator of BDNF in Mouse Cortical Neurons

**DOI:** 10.3390/ijms23126853

**Published:** 2022-06-20

**Authors:** Mona Abdollahi, Margaret Fahnestock

**Affiliations:** 1 Medical Sciences Graduate Program, Faculty of Health Sciences, McMaster University, 1280 Main Street West, Hamilton, ON L8S 4K1, Canada; monaa@mcmaster.ca; 2 Department of Psychiatry and Behavioral Neurosciences, McMaster University, 1280 Main Street West, Hamilton, ON L8S 4K1, Canada

**Keywords:** NR4A2/Nurr1, BDNF, membrane depolarization, amodiaquine, Nurr1 siRNA

## Abstract

Nurr1 and brain-derived neurotrophic factor (BDNF) play major roles in cognition. Nurr1 regulates *BDNF* in midbrain dopaminergic neurons and cerebellar granule cells. Nurr1 and BDNF are also highly expressed in the cerebral cortex, a brain area important in cognition. Due to Nurr1 and BDNF tissue specificity, the regulatory effect of Nurr1 on *BDNF* in different brain areas cannot be generalized. The relationship between Nurr1 and BDNF in the cortex has not been investigated previously. Therefore, we examined Nurr1-mediated *BDNF* regulation in cortical neurons in activity-dependent and activity-independent states. Mouse primary cortical neurons were treated with the Nurr1 agonist, amodiaquine (AQ). Membrane depolarization was induced by KCl or veratridine and reversed by nimodipine. AQ and membrane depolarization significantly increased *Nurr1* (*p* < 0.001) and *BDNF* (*p_AQ_* < 0.001, *p_KCl_* < 0.01) as assessed by real-time qRT-PCR. However, *Nurr1* knockdown did not affect *BDNF* gene expression in resting or depolarized neurons. Accordingly, the positive correlation between *Nurr1* and *BDNF* expression in AQ and membrane depolarization experiments does not imply co-regulation because *Nurr1* knockdown did not affect *BDNF* gene expression in resting or depolarized cortical neurons. Therefore, in contrast to midbrain dopaminergic neurons and cerebellar granule cells, Nurr1 does not regulate *BDNF* in cortical neurons.

## 1. Introduction

The nuclear receptor subfamily 4 group A member 2 (NR4A2, also known as nuclear receptor-related 1 protein or Nurr1, NOT, TINUR, NGFIB, HZF-3, and RNR1) is an orphan receptor and a transcription factor that belongs to the class of steroid nuclear hormone receptors [1]. Nurr1 is well known for playing a fundamental role in the regulation of the development and maintenance of midbrain dopaminergic (mDA) neurons [2]. Nurr1 binds to brain-derived neurotrophic factor (*BDNF*) promoters in mDA neurons and cerebellar granule cells (CGCs) [3,4,5,6]. BDNF plays a pleiotropic role in the central nervous system. BDNF promotes the survival of mDA neurons, and its deprivation leads to cell death [7]. However, neither Nurr1 nor BDNF expression and roles are limited to mDA neurons. BDNF is highly expressed in the hippocampus and cortex [8]. The communication between the hippocampus and the cerebral cortex plays a leading role in complex cognitive functions, including memory [9,10,11,12]. In cortical neurons, BDNF regulates dendrite growth [13], development [14], plasticity [15,16], survival [17,18], differentiation [19], and neural circuit formation [20]. These processes underlie the mechanisms of learning and memory. Cognitive decline, especially memory deficits, is the hallmark of dementia and is tightly associated with reduced expression of cortical BDNF [21,22]. *BDNF* is differentially regulated in response to internal and external stimuli that impact neuronal activity [23]. As a result, the identification of transcription factors that regulate *BDNF* in a tissue-and activity-dependent manner provides a better understanding of the underlying molecular mechanisms.

Nurr1 regulates *BDNF* in mDA and CGCs and may also do so in cerebral cortical neurons. Nurr1 is highly expressed in cortical regions [11,12]. In addition, similar to BDNF, Nurr1 and its transcriptional targets play critical roles in cortical-dependent cognition, especially learning and memory [12,24,25,26,27,28,29,30,31,32,33,34]. Nurr1 repression contributes to age-related impairments in memory [35,36,37,38,39,40,41] and Alzheimer’s disease, the most common form of dementia [42]. However, the role of Nurr1 in the regulation of *BDNF* has not been investigated in this brain region. Furthermore, due to BDNF tissue specificity [43,44], the regulation of *BDNF* by Nurr1 in one region of the brain cannot be generalized to other areas. As a result, we investigated whether Nurr1 regulates *BDNF* expression in mouse cortical neurons in vitro. 

## 2. Results

### 2.1. Pharmacological Stimulation of Nurr1 Increases Nurr1 and BDNF Gene Expression

**Figure 1:**
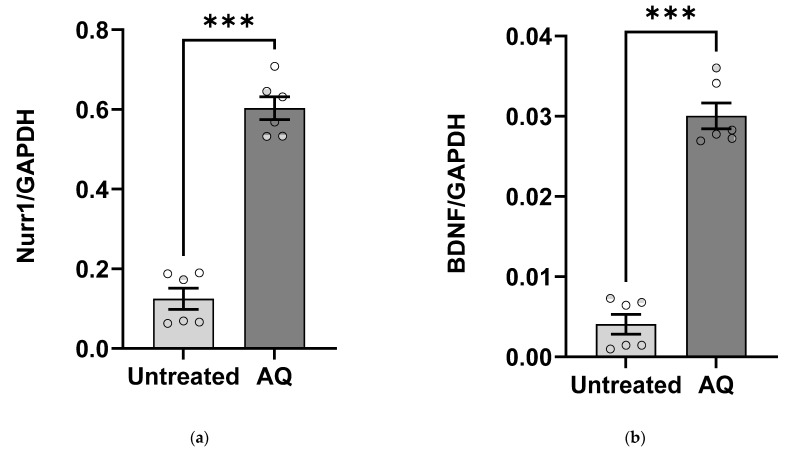
AQ treatment increases *Nurr1* and *BDNF* gene expression. Primary cortical neurons were treated with 10 µM AQ for 24 h. Real-time qRT-PCR data shows that compared to untreated cells, AQ-treated cells exhibited significantly increased levels of (**a**) Nurr1 and (**b**) BDNF mRNA. Both targets were normalized to the housekeeping gene GAPDH, which did not change upon AQ treatment (*p* > 0.05). Two-way ANOVA and *post hoc* independent samples *t*-test, *** *p* < 0.001, *n* = 6 per group. Error bars: ±*SE*.

We investigated the changes in *BDNF* gene expression in response to the Nurr1 agonist amodiaquine (AQ). A two-way ANOVA for *Nurr1* and *BDNF* (experiment × treatment) showed a main effect of treatment for both targets (*p* ˂ 0.001). There was no main effect of experiment (*p_Nurr1/GAPDH_* = 0.11, *p_BDNF/GAPDH_* = 0.16) or interaction between experiment and treatment (*p_Nurr1/GAPDH_* = 0.11, *p_BDNF/GAPDH_* = 0.16), so experiments were combined for further analysis. *Post hoc* independent samples t-test revealed that AQ treatment significantly increased Nurr1 (Figure 1a, *p* ˂ 0.001) and BDNF (Figure 1b, *p* ˂ 0.001) mRNA expression compared to untreated cells. 

### 2.2. Membrane Depolarization Induces Nurr1 and BDNF Gene Expression

**Figure 2:**
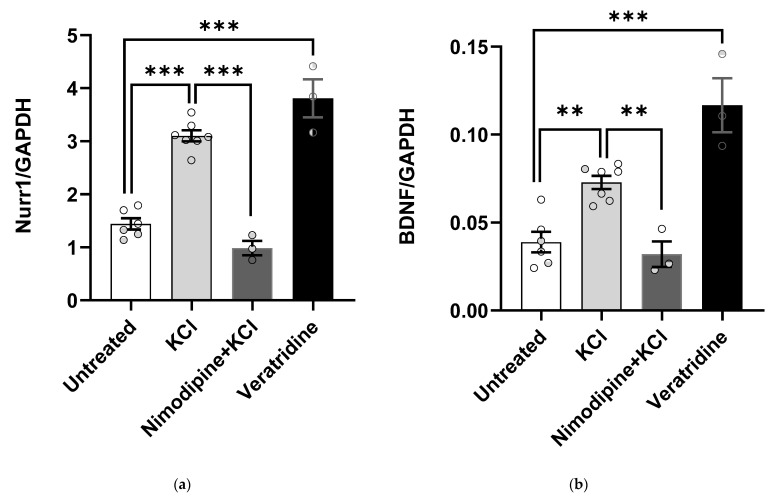
Membrane depolarization increases *Nurr1* and *BDNF* gene expression. Primary cortical neurons were treated with 50 mM KCl with or without 10 µM nimodipine or with 10 µM veratridine as described in Methods. Compared to untreated cells (*n* = 6), KCl- (*n* = 7) and veratridine-treated cells (*n* = 3) exhibited significantly increased levels of (**a**) Nurr1 and (**b**) BDNF mRNA. Nimodipine (*n* = 3) blocked the increase in Nurr1 (*p* = 0.405) and BDNF mRNA (*p* = 0.98). Two-way ANOVA and *post hoc* Gabriel’s test, ** *p* < 0.01 and *** *p* < 0.001. Error bars: ±*SE*.

To understand the effect of Nurr1 on *BDNF* regulation during neuronal activity, we applied KCl-mediated membrane depolarization to primary cortical cell cultures. Veratridine was used as a positive control for membrane depolarization [43,44,45], and Nimodipine was used as negative control [46]. A two-way ANOVA for *Nurr1* and *BDNF* expression (experiment x treatment) revealed a main effect of treatment (*p* ˂ 0.001). There was no statistically significant interaction between the effects of experiment and treatment (*p_Nurr1/GAPDH_* = 0.85, *p_BDNF/GAPDH_* = 0.70) and no main effect of experiment (*p_Nurr1/GAPDH_* = 0.97, *p_BDNF/GAPDH_* = 0.30), so experiments were combined for further analysis. *Post hoc* Gabriel’s test indicated that, compared to either untreated cells or KCl-induced cells pretreated with nimodipine, both KCl and veratridine significantly induced *Nurr1* (*p* < 0.001, Figure 2a). KCl significantly increased *BDNF* gene expression compared to untreated cells (*p* = 0.009) and depolarized cells that were pre-treated with nimodipine (*p* < 0.001). Likewise, veratridine treatment induced *BDNF* expression compared to both untreated (*p* = 0.01) and nimodipine (*p* < 0.001) groups (Figure 2b). 

### 2.3. Basal BDNF Gene Expression Is Not Regulated by Nurr1

**Figure 3:**
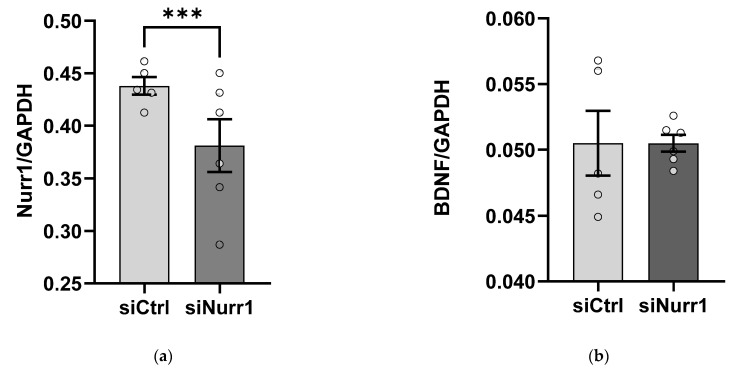
Nurr1 siRNA knockdown does not alter *BDNF* gene expression. Primary cortical cell cultures were treated with 35 pmol scrambled siRNA or Nurr1 siRNA and 48 h later were harvested for real-time qRT-PCR. (**a**) Compared to the control group, Nurr1 mRNA was reduced significantly in the siNurr1 group. (**b**) BDNF mRNA remained unchanged between groups. Two-way ANOVA and *post hoc* independent samples *t*-test, *** *p* < 0.001, *n* = 5 per group. Error bars: ±*SE*.

We examined the effect of partial loss of Nurr1 on *BDNF* gene expression in cortical neurons. We knocked down *Nurr1* in primary cortical neurons using siRNA and monitored subsequent changes in *BDNF* gene expression with real-time qRT-PCR. We ruled out the possibility of toxic effects of Nurr1 siRNA under our experimental conditions because (1) microscopic evaluation and LDH assay showed no signs of toxicity and (2) GAPDH gene expression remained unchanged in Lipofectamine control, scrambled siRNA, and Nurr1 siRNA conditions throughout all experiments (data not shown). A two-way ANOVA (experiment × treatment) showed that there was a main effect of treatment on Nurr1 mRNA levels (*p* < 0.001), no main effect of the experiment (*p* = 0.19), and no statistically significant interaction between the effects of experiment and treatment (*p* = 0.63). As a result, experiments were combined for further analysis. A *post hoc* t-test showed that Nurr1 mRNA was reduced by 27.6% ± 0.023 in the siNurr1 group compared to the siCtrl group (*p* < 0.001, Figure 3a). In contrast, no statistically significant difference was found in BDNF mRNA levels between siNurr1 and siCtrl groups (two-way ANOVA *p*_experiment_ = 0.84, *p*_treatment_ = 0.086, *p*_experiment_ _× treatment_ = 0.22, Figure 3b). 

### 2.4. Activity-Dependent BDNF Gene Expression Is Not Regulated by Nurr1

**Figure 4:**
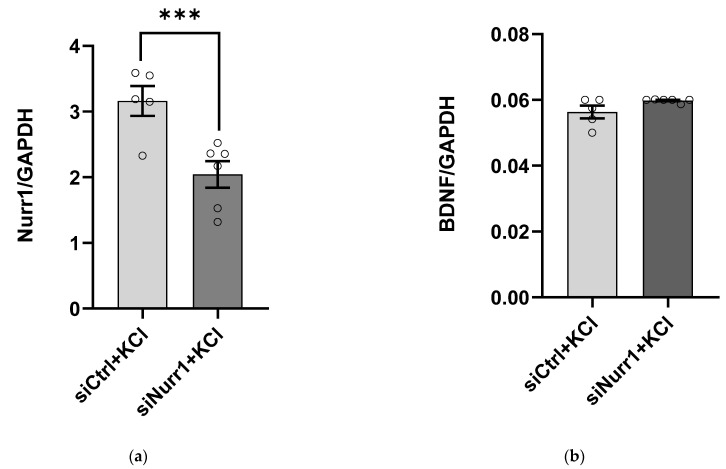
*Nurr1* knockdown does not affect activity-dependent *BDNF* gene expression. Primary cortical neurons were treated with 50 mM KCl and 35 pmol of scrambled siRNA or Nurr1 siRNA. Cells were harvested forty-eight hours post-transfection and underwent harvest for real-time qRT-PCR. (**a**) Nurr1 mRNA was reduced significantly in the siNurr1 group compared to the control group. (**b**) BDNF mRNA remained unchanged between groups. *** *p* < 0.0001, two-way ANOVA and *post hoc* independent samples *t*-test (*n* = 5–6 per group). Error bars: ±*SE*.

To understand the role of Nurr1 in *BDNF* regulation during neuronal activity, we applied KCl to induce membrane depolarization in primary cortical cell cultures. A two-way ANOVA (experiment × treatment) showed that there was a main effect of treatment (*p* = 0.001) on Nurr1 mRNA levels. No main effect of the experiment (*p* = 0.28) and no statistically significant interaction between the effects of the experiment and treatment (*p* = 0.058) was detected. As a result, results from three separate experiments were combined for further analysis. Our results revealed that *Nurr1* gene expression was knocked down by 35.4% ± 0.43 in KCl-induced cortical neurons (*post hoc* independent samples t-test, *p* = 0.005, Figure 4a). For *BDNF* expression, two-way ANOVA showed no main effect of experiment (*p* = 0.77), no main effect of treatment (*p* = 0.18), and no interaction between experiment and treatment (*p* = 0.96, Figure 4b).

## 3. Discussion

Regulation of *BDNF* by Nurr1 in cortical neurons is not supported by our Nurr1 knockdown experiments, in which Nurr1 knockdown did not affect *BDNF* expression. Therefore, the increase observed in BDNF mRNA following KCl and AQ treatment does not indicate a causative relationship, but rather a positive correlation. It is reasonable to speculate that increased BDNF mRNA in response to AQ treatment and membrane depolarization is a consequence of the downstream recruitment of other transcriptional regulators/co-regulators and/or dissociation of repressors rather than an off-target effect. We chose to treat our cells with AQ for two main reasons. First, Nurr1 activation by AQ has been linked to neuroprotection. Activation of Nurr1 by AQ leads to the generation of neurons from cortical [42] and hippocampal [42,47,48] neural precursor cells, enhances short-and long-term memory, and improves cognitive deficits in mouse models of Parkinson’s [49,50] and Alzheimer’s disease [51]. Second, AQ is known to be a selective agonist for Nurr1 with no effect on other members of the NR4A family (Nur77 and NOR1) [49]. However, it has been shown by a recent study that a high concentration of AQ (100 µM) which is above the EC50 (36 ± 4 µM) activates Nur77 and NOR1 in cell-free experiments [52]. 

We intentionally used 10 µM AQ in our study to avoid off-target effects of AQ. Therefore, it is unlikely that increased BDNF mRNA is a consequence of Nurr1-independent AQ effects. On the other hand, nuclear receptors such as Nurr1, acting either in combination or sequentially, can recruit core transcription factors, RNA polymerase II, and chromatin remodeling factors to specific promoters [53]. Nurr1 AQ-dependent recruitment of such transcriptional coregulators has been shown in SK-N-BE (2)C cells, a human neuroblastoma cell line [49]. Furthermore, increased Nurr1 mRNA levels suggest the presence of a positive feedback loop. The formation of positive feedback loops by Nurr1’s gene targets and downstream signaling has been shown in previous studies [54,55].

One important tissue-specific feature of neuronal activity is its involvement in synaptic plasticity via the regulation of neurotrophins, particularly BDNF [56,57]. Our data are in agreement with previous studies that show *Nurr1* and *BDNF* gene expression are induced in depolarized primary neural cultures [3,4,17,46,58,59,60,61]. However, similar to AQ treatment experiments, the nature of membrane depolarization experiments does not demonstrate a direct regulatory role of Nurr1 on *BDNF* because *Nurr1* and *BDNF* are both immediate/early genes that can be induced downstream of membrane depolarization as components of unrelated molecular pathways [62,63,64,65]. 

Cortical Nurr1 and BDNF are reduced in neurological disorders exhibiting impairment [66,67]. To mimic Nurr1 reduction, we used Nurr1 siRNA. When Nurr1 is knocked down by siRNA, BDNF mRNA expression does not change either in basal or activity-induced conditions. Signaling pathways that are triggered by membrane depolarization and lead to Nurr1 induction are cell- and tissue-specific. *Nurr1* and *BDNF* upregulation in induced mDA neurons are PKC/PLC-dependent [3], while NMDA receptors play a key role in the upregulation of Nurr1 and BDNF in CGCs [58]. In contrast, AMPA- and NMDA-type glutamate receptors do not play a role in Nurr1 induction in hippocampal and cortical neurons [46]. It has been shown that in depolarized hippocampal and cortical neurons, upregulation of *Nurr1* is mediated by calcineurin [46]. Calcineurin can activate CREB, a well-known regulator of *Nurr1* and *BDNF* [68,69,70]. This suggests that in response to membrane depolarization in cortical neurons, activated CREB might be responsible for the enhancement of its target genes, *Nurr1* and *BDNF*. 

## 4. Materials and Methods

### 4.1. Neuronal Cell Culture

Cell culture reagents and media were purchased from ThermoFisher Scientific, Burlington, Ontario, Canada. One day before brain dissection, cell culture plates were coated with 1 mL poly-L-lysine. Wells were washed once with phosphate-buffered saline and preconditioned with 1 mL Neurobasal™ medium for at least one hour before cell culture. Primary cell cultures of cortical neurons were prepared from embryonic day 17–18 (E17-18) C57Bl/6 mice as described previously [69]. For AQ treatment and membrane depolarization experiments isolated cortical neurons were seeded at a density of 10^6^ cells/well in 6-well plates. For transfection experiments, 10^4^ cells/well were seeded in 12-well plates. Prepared cell cultures were incubated at 37 °C and 5% CO_2_. The cell culture medium consisted of 2% B-27 supplement, 1% Penicillin-Streptomycin, 1% GlutaMAX™ supplement, and 1% fetal bovine serum (FBS) in Neurobasal™ medium. After 24 h, the cell culture medium was replaced by a maintenance medium (cell culture medium lacking FBS). Half of the medium in each well was replaced by an equal amount of fresh maintenance medium every 72 h. 

### 4.2. RNA Extraction

Primary cortical neurons were harvested in 1ml TRIzol^TM^ reagent per well. RNA was isolated using PureLink™ RNA Micro with on-column DNAse treatment (ThermoFisher Scientific, Burlington, ON, Canada) according to the manufacturer’s instructions. RNA yield, purity, and integrity were determined by spectrophotometry and agarose gel electrophoresis.

### 4.3. Amodiaquine (AQ) Treatment

AQ stock solution (Sigma-Aldrich, Burlington, ON, Canada) was prepared in water (10 mM). At 7 days in vitro (DIV7), primary cortical neurons were treated with a maintenance medium containing 10 µM AQ. Cells were harvested for RNA extraction 24 h later. 

### 4.4. Membrane Depolarization

Veratridine (Sigma-Aldrich, Burlington, ON, Canada) stock solution (37 mM) was prepared in DMSO. At DIV7, cortical cell cultures were depolarized in a maintenance medium containing 50 mM KCl or 10 µM veratridine for 2 h and then incubated without KCl or veratridine for another 2.5 h before harvesting for RNA extraction. A Nimodipine (Sigma-Aldrich, Burlington, ON, Canada) stock solution (50 mM) was prepared in DMSO. Neurons were pre-treated with 10 µM nimodipine for 10 min before stimulation with 50 mM KCl. The final concentration of DMSO used as a vehicle was less than 0.01% (*v*/*v*). 

### 4.5. Nurr1 Knockdown

Lipofection reagents and media were purchased from ThermoFisher Scientific, Burlington, Ontario, Canada. Cortical neurons were transfected with Silencer™ Select pre-designed murine Nurr1 siRNA (siRNA ID# s70890) at DIV4 in an antibiotic-free maintenance medium. The final concentration of siRNA used per well was 35 pmol and the final volume of Lipofectamine™ RNAiMAX used per well was 1.75 µL. The siRNA-Lipofectamine complex was incubated at room temperature for 5 min before adding to cortical neurons. Cells were harvested 48 h after incubation at 37 °C and 5% CO_2_ for RNA extraction. For Nurr1 knockdown experiments in KCl-induced cortical neurons, cells were first transfected as above. After 48 h, the transfection medium was replaced with antibiotic-free maintenance medium containing 50 mM KCl for 2 h, followed by 2.5 h in an antibiotic-KCl-free maintenance medium before harvesting for RNA extraction.

### 4.6. Complementary DNA (cDNA) Synthesis

Reagents used for cDNA synthesis were obtained from ThermoFisher Scientific, Burlington, Ontario, Canada. The reaction mixture for cDNA synthesis (total volume of 13 µL) consisted of 1 µg RNA, 1 µL dNTP mixture (10 mM of each of dATP, dGTP, dCTP, and dTTP), and 1 µL (300 ng/μL) of random primers. After incubation at 65 °C for 5 min, 7 µL of master mix including dithiothreitol (100 mM), RNaseOUT (40 units), and SuperScript^TM^ III or IV (200 units) in 5× First-Strand Buffer or 5× SuperScript IV buffer was added to each sample to a total volume of 20 µL. A no-reverse-transcriptase negative control lacked SuperScript^TM^ III or IV and instead, 1µL of ddH_2_O was added. The reaction was run in a GeneAmp PCR system 2400 thermal cycler (Applied Biosystems, Foster City, CA, USA) at 25 °C for 5 min, followed by 50 °C for 50 min, and heat inactivation at 70 °C for 15 min (SuperScript^TM^ III) or at 23 °C for 10 min, followed by 53 °C for 10 min, and heat inactivation at 80 °C for 10 min (SuperScript^TM^ IV). 

### 4.7. Real-Time Quantitative Reverse Transcription-Polymerase Chain Reaction (qRT-PCR)

Reagents used for gene expression analysis were obtained from ThermoFisher Scientific, Burlington, Ontario, Canada. Primers used were Nurr1 primers [GenBank: *NM_013613.2*] (sense: 5′-CAA CTA CAG CAC AGG CTA CGA-3′ and antisense: 5′-GCA TCT GAA TGT CTT CTA CCT TAA TG-3′, Mobix, Hamilton, ON, Canada), *BDNF* primers [GenBank: NM 001048139.1] (sense: 5′-GCG GCA GAT AAA AAG ACT GC-3′, antisense: 5′-CTT ATG AAT CGC CAG CCA AT-3′, Mobix) and *GAPDH* primers [GenBank: NM 001289726.1] (sense: 5′-GTG GAG TCA TAC TGG AAC ATG TAG-3′, and antisense: 5′-AAT GGT GAA GGT CGG TGT G-3′, Mobix). Standards for absolute quantification were prepared using the above target-specific primers as described previously [71]. Real-time amplifications were carried out in triplicate with QuantStudio™3 Real-Time PCR System (Thermo Fisher Scientific Inc., Waltham, MA, USA). Samples lacking reverse transcriptase were used as negative controls to confirm the lack of genomic DNA contamination. The thermal profile for *Nurr1* was 2 min at 50 °C, 2 min at 95 °C, followed by 40 cycles of 95 °C for 15 sec, 60 °C for 1 min. The thermal profile for *BDNF* and *GAPDH* was 2 min at 50 °C, 2 min at 95 °C followed by 40 cycles of 95 °C for 30 s, 58 °C for 30 s, and 72 °C for 45 s. A dissociation curve was followed to monitor the formation of any secondary products. Nurr1 and BDNF mRNA quantities are presented as a ratio to copy numbers of *GAPDH*, which did not change with any treatment. mRNA quantities and PCR efficiencies were calculated using QuantStudio™ Design and Analysis Software (Version 1.5.1, Thermo Fisher Scientific, Inc.).

### 4.8. Statistical Analysis 

Statistical analyses were performed using SPSS software (Version 26, IBM Corp, Armonk, NY, USA). The homogeneity of variances was assessed by Levene’s test. Shapiro-Wilk test of normality was used to test the normal distribution of data. When data departed from normality, a combination of calculated z scores for skewness and kurtosis and visual inspection was used to decide whether the assumption of normality was acceptable or not. Further, the overall assumption was that parametric tests, including t-test (assuming equal variances) and univariant analysis of variance (ANOVA), were robust to moderate departures from normality and homogeneity. All experiments were conducted on 2–4 samples in each of two separate experiments. A two-way ANOVA between groups and experiments was used to verify that no significant differences occurred between the two experiments. *Post hoc* comparisons included unpaired, two-tailed Student’s t-tests and Gabriel’s tests (for unequal sample sizes). *p* ≤ 0.05 was considered statistically significant. GraphPad Prism (Version 9.0.0, GraphPad Software, LCC, San Diego, CA, USA) was used to generate graphs.

## 5. Conclusions

In summary, we showed that *BDNF* expression is regulated independently of Nurr1 in cortical neurons. Tissue-specific *BDNF* regulation by Nurr1 may require molecular components that are absent in cortical neurons but are present in mDA neurons and CGCs. Our findings highlight the importance of studying *BDNF* transcriptional regulation in different tissues.

## Data Availability

The data presented in this study are openly available in Scholars Portal Dataverse at https://doi.org/10.5683/SP3/WXDUBJ (accessed on 7 June 2022).
